# Enhanced performance of the microalga *Chlorella sorokiniana* remotely induced by the plant growth-promoting bacteria *Azospirillum brasilense* and *Bacillus pumilus*

**DOI:** 10.1038/srep41310

**Published:** 2017-02-01

**Authors:** Edgar Amavizca, Yoav Bashan, Choong-Min Ryu, Mohamed A. Farag, Brad M. Bebout, Luz E. de-Bashan

**Affiliations:** 1 Environmental Microbiology Group, Northwestern Center for Biological Research (CIBNOR), Av IPN 195, La Paz, B.C.S. 23096, Mexico; 2 The Bashan Institute of Science, 1730 Post Oak Court, Auburn AL 36830, USA; 3 Dept. of Entomology and Plant Pathology, 301 Funchess Hall, Auburn Univ., Auburn, AL 36849, USA; 4 Molecular Phytobacteriology Laboratory, Korean Research Institute of Bioscience and Biotechnology (KRIBB), Daejeon 305-600, South Korea; 5 Department of Pharmacognosy, Faculty of Pharmacy, Cairo University, Cairo 11562, Egypt; 6 Exobiology Branch, NASA Ames Research Center, Moffett Field, CA 94035, USA

## Abstract

Remote effects (occurring without physical contact) of two plant growth-promoting bacteria (PGPB) *Azospirillum brasilense* Cd and *Bacilus pumilus* ES4 on growth of the green microalga *Chlorella sorokiniana* UTEX 2714 were studied. The two PGPB remotely enhanced the growth of the microalga, up to six-fold, and its cell volume by about three-fold. In addition to phenotypic changes, both bacteria remotely induced increases in the amounts of total lipids, total carbohydrates, and chlorophyll *a* in the cells of the microalga, indicating an alteration of the microalga’s physiology. The two bacteria produced large amounts of volatile compounds, including CO_2_, and the known plant growth-promoting volatile 2,3-butanediol and acetoin. Several other volatiles having biological functions in other organisms, as well as numerous volatile compounds with undefined biological roles, were detected. Together, these bacteria-derived volatiles can positively affect growth and metabolic parameters in green microalgae without physical attachment of the bacteria to the microalgae. This is a new paradigm on how PGPB promote growth of microalgae which may serve to improve performance of *Chlorella* spp. for biotechnological applications.

*In vitro* culturing at laboratory or mass industrial scales is the most common way by which the biotechnological industry is producing products from microalgae^1^,^2^ and inoculants of plant growth-promoting bacteria (PGPB) for agricultural and environmental applications^3^. The current paradigm of how PGPB enhance plant growth is through attachment of the bacteria to plant roots. PGPB first establish a stable physical interaction with its host and subsequently colonize the root system, which results in beneficial effects on plants^4^,^5^ When the PGPB move toward its host before attachment, the chances of successful colonization improve^6^. In the absence of attachment and colonization to plant surface, no effect of PGPB on higher plants are recorded^7^.

Attachment of the PGPB *Azospirillum* spp. to roots of many plant species has been demonstrated since the emergence of the field some 40 years ago^4^,^8^,^9^,^10^,^11^,^12^. *A. brasilense* has also been observed to attach to, and form stable aggregates with, the microalga *Chlorella vulgaris*^13^. Similar interactions have also been proposed for Gram-positive bacteria, such as *Bacillus* spp.^14^. While attachment to roots has been documented as the major requirement for a beneficial association of PGPB with plants^7^, it is also well documented that the PGPB *Azospirillum* spp. can enhance many biotechnological processes of interest through association with the microalgae *Chlorella* spp. Processes enhanced by the association of *Azospirillum* with *Chlorella* spp. include: production of carbohydrates^15^,^16^, lipids and fatty acids^17^, photosynthetic pigments^18^, algal biomass^19^,^20^, C and N transport^21^. Several of the metabolic processes enhanced by this association have been applied to wastewater treatment^22^. All of these processes were demonstrated to be enhanced under the conditions in which *Azospirillum* was kept in close proximity or actually attached to the *Chlorella* spp.^23^ and they are attributed to transfer of metabolites, mainly the phytohormone indole-3-acetic acid (IAA) from the bacteria to the microalgae^24^,^25^. However, the requirement of a firm attachment between the two microorganisms has not been demonstrated as it has in higher plants.

Production of volatile compounds by microorganisms commonly occurs as part of normal metabolism, and plays a role in the communication within, and between, organisms. Plants respond to these volatiles both by increasing and reducing growth^26^,^27^. Volatiles emitted by PGPB^28^,^29^, such as 3-hydroxy-2-butanone (acetoin) and 2,3-butanediol, produced by *Bacillus* spp. and *Burkholderia* spp., have been shown to stimulate the growth of *Arabidopsis thaliana* without physical contact^30^,^31^. Beneficial microbial volatile organic compounds (mVOCs) have been reported in several model plants, including peppermint^32^, alfalfa (lucerne)^33^, tobacco^34^, and *Physcomitrella* moss^35^. In contrast, several bacterial volatiles, such as ammonia^36^, ethylene^37^, and hydrogen cyanide^38^ can harm plants. While more than 300 potential molecules with the capacity to affect plants have been identified^27^,^30^,^39^,^40^, GC-MS analyses of bacterial volatiles continue to reveal many compounds that have yet to be identified as serving a function in plants. Positive effects, well-demonstrated in assays using miniature laboratory plant models grown in Petri dishes, are significantly more challenging to demonstrate with full-sized plants^41^.

Although *Chlorella* spp. are found in natural bodies of water, these microalgae are commonly cultured for biotechnological purposes^2^. Cultures of *Chlorella* spp. provide a convenient tool to determine whether volatiles produced by PGPB can affect plant cells in the absence of physical attachment. *Azospirillum brasilense* is a common diazotrophic PGPB that fixes nitrogen only under microaerophilic conditions. It affects the growth of *Chlorella* spp. via direct diffusion of IAA, a non-volatile crystalline compound from cell to cell. These two growth conditions were not available for the microalgae in our study where signal transmission between the bacteria and the microalgae was solely by volatile compounds. *Bacillus pumilus* is a PGPB, originally isolated from a cactus, which affects growth of *C. vulgaris* when co-immobilized in alginate beads^42^ via a currently unknown mechanism.

We test here the hypothesis that *Azospirillum brasilense* and *Bacillus pumilus*, can remotely affect the growth and metabolism of the microalgae *C. sorokiniana* from PGPB volatiles. To test our hypothesis, we developed an *in vitro* system in which the microorganisms were cultured separately (without physical contact, and allowing only the exchange of volatiles). We measured microalgal growth and three major metabolic products (carbohydrates, lipids, and chlorophyll *a*), which have previously been shown to be enhanced by these PGPB when attached to the microalgae^43^. This investigation was intended to determine whether it is possible to promote the growth of microalgae from bacterial volatiles and, if so, whether this approach could be a tool for biotechnological applications of microalgae.

## Results

### Promotion of algal growth by airborne microbial volatiles from PGPB

**Figure 1:**
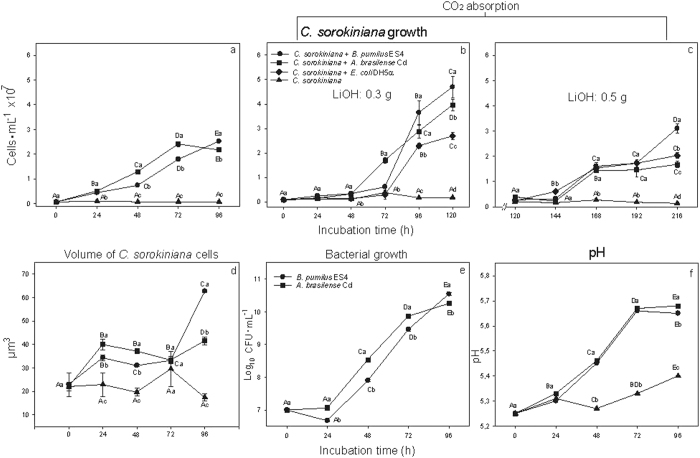
(**a**) Remote effect of emissions of the PGPB *Azospirillum brasilense* Cd and *Bacillus pumilus* ES4 and the control bacterium *Escherichia coli* DH5α on growth of the microalgae *Chlorella sorokiniana*, (**b**) growth in the presence of the CO_2_ absorbant LiOH•H_2_O, 0.3 g and (**c**) 0.5 g. (**d**) Remote effect of emissions on the volume of *C. sorokiniana* cells, (**e**) Growth of the PGPB in the culture medium, and (**f**), Increase in pH in the medium. The control bacterium (*E. coli*) was used only in experiments measuring the potential effect of produced CO_2_ on microalgae growth and metabolite production. Values on curves denoted by different capital letters differ significantly using one-way ANOVA combined with LSD post-hoc analysis at *P* < 0.05. Points at each time interval denoted by different lowercase letters differ significantly at *P* < 0.05 in (**a–d, f**) using ANOVA and in (**e**) using Student’s *t*-test).

Both PGPB significantly enhanced growth of *C. sorokiniana* remotely up to six-fold, relative to the controls, in four days (Fig. 1a). Initial enhancement during the first three days was higher with *A. brasilense*; after four days, enhancement of population growth with *B. pumilus* was higher (Fig. 1a). Reducing CO_2_ in the atmosphere of the Kitasato flask by incorporating a filter made of lithium hydroxide completely stopped growth of the microalgae over short and long time intervals, as expected (Fig. 1b,c). However, with the two PGPB located remotely and also when adding the control bacterium *Escherichia coli* to the experimental setup, promotion of growth occurred when the incubation period was extended. Cell volume increases were also observed in algal cells remotely connected to both PGPB, whereas microalgae cultured alone did not increase in cell volume (Compare Fig. 1a and Fig. 1d).

### Production of CO_2_ and mVOC by PGPB

Both PGPB grew well in Brunner’s medium (Fig. 1e), and both bacteria increased the pH of their growth medium in a similar manner (Fig. 1f). After two days in Brunner’s medium, both PGPB produced significant amounts of CO_2_, as expected, which accumulated in the headspace at concentrations significantly higher than atmospheric CO_2_ (Table 1). *B. pumilus* produced a larger amount of CO_2_ with fewer cells than *A. brasilense* (Table 1). *E. coli* is well known to produce significant amounts of CO_2_ under these conditions^44^. SPME coupled to GC-MS analysis identified 47 volatiles produced by PGPB (Table 2). A representative gas chromatogram is shown in Suppl. Fig. 1, showing the differences in volatile emission patterns between strains of *A. brasilense, B. pumilus*, and *E. coli*. GC-MS analysis of mVOCs revealed consistent differences in the volatile blends released by specific strains of *A. brasilense* and *B. pumilus*, and to *E. coli* (Table 2, mass spectra of major labeled peaks are presented as Supplementary Material, Fig. S4). In all cases, reported values represent the relative percentile of each percentage of total volatile to the total amount of volatiles detected with volatile peak area being first normalized to the amount of the spiked internal standard hexneyl acetate. No absolute measurements of individual of volatiles were made considering that no standard calibration curve was made for each target volatile with its standard. Such an approach has been reported from our work reporting plant volatiles analysis using headspace SPME^45^. Growth-promoting volatiles 2,3-butanediol and 3-hydroxy-2-butanone (also referred to as acetoin) were consistently major volatile components (75% and 62% of total volatile blend) produced by *A. brasilense* and *B. pumilus*, respectively, whereas these volatiles were released at much lower levels by *E. coli* (4%). 3-methyl-1-butanoic acid (isovaleric acid), 2-methyl-butyric acid, and 3-methyl-1-butanol (also referred to as fusel alcohol) were found only in *A. brasilense,* and *B. pumilus*. Several short-chain fatty acyl esters (octanoic, decanoic, and dodecanoic acid ethyl esters) were identified, though at much lower levels compared to short-chain alcohols in *A. brasilense* and *B. pumilus* volatile blends.

### A remote effect induced by the PGPB and *E. coli* on accumulation of total lipids, carbohydrates, and chlorophyll a *in C. sorokiniana*

**Figure 2:**
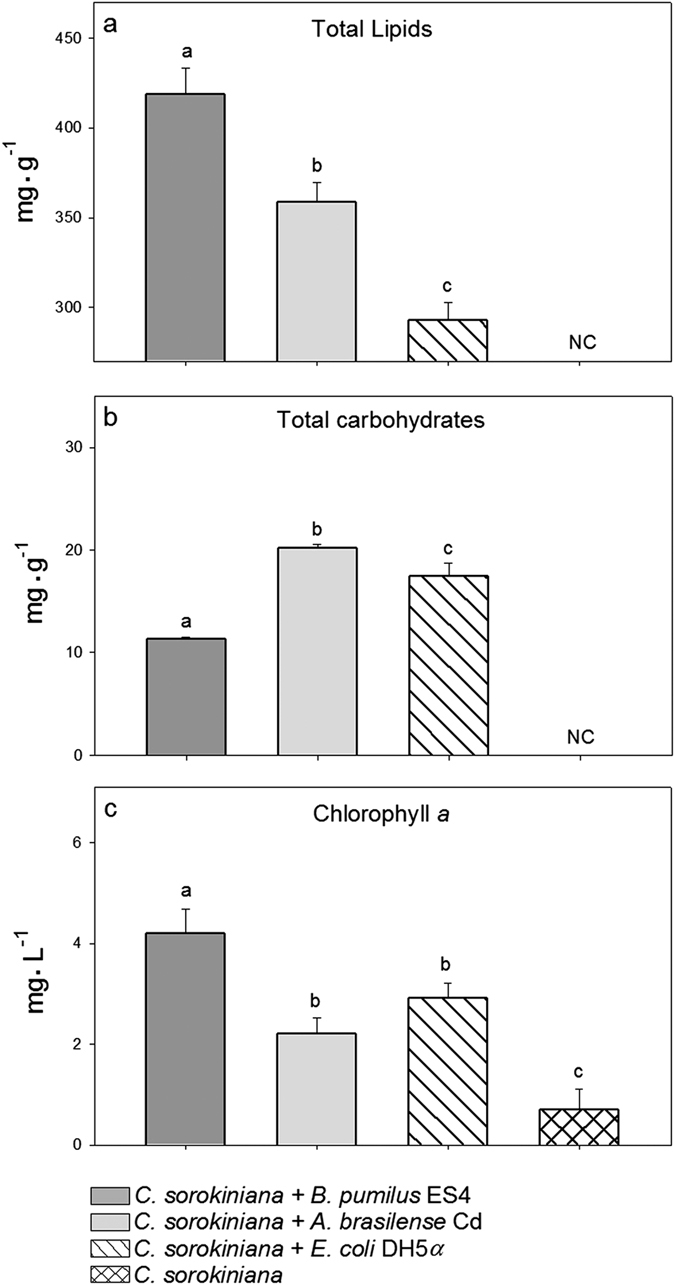
Remote effects of emissions on accumulation of lipid, carbohydrate, and chlorophyll *a* content in *Chlorella sorokiniana* by the PGPB *Azospirillum brasilense* Cd and *Bacillus pumilus* ES4 after 96 h of incubation. In each subfigure, columns denoted by different letters are significantly different. Analyses were made by one-way ANOVA and LSD post-hoc analysis at *P* < 0.05.

Total lipids were significantly enhanced by a remote effect from all three species of bacteria (two PGPB and the control *E. coli*), with *B. pumilus* inducing the highest effect. Lipids produced by microalgae growing alone were below the detection limit of the method used (Fig. 2a). Similar results occurred for carbohydrate production by the microalgae, but in this case, *A. brasilense* induced the highest effect (Fig. 2b). All bacterial species enhanced the quantity of chlorophyll *a*, relative to the level of chlorophyll *a* in microalgae growing alone (Fig. 2c).

## Discussion

Culturing microalgae in the presence of PGPB is an artificially created experimental situation. For biotechnological purposes, any beneficial interaction occurring under these artificial conditions would result in an economic gain and therefore be worth exploring. The main purpose of this proof-of-concept study was to show that the enhancement of performance of microalgae, by volatiles of PGPB, is an effective strategy for common biotechnological applications involving cellular growth and metabolite production. As our results demonstrate, the enhancement of algal production by PGPB can be accomplished without attachment of the PGPB to plant surfaces (as normally occurs when PGPB is inoculated on plants).

The significant positive effects of both *A. brasilense* and *B. pumilus* on general growth and metabolism of *Chlorella* spp. when the microorganisms are in forced close proximity inside an alginate bead is well documented (see Introduction for references). At least one genus of PGPB, *Bacillus,* was known to produce a variety of volatiles^27^. Both PGPB are expected to produce CO_2_ during normal aerobic respiration, the growth conditions used in this study. Consequently, it is possible that the effects of volatiles will affect performance of microalgae.

The design of the experiments presented here ensures a lack of physical contact between any PGPB and the microalgae. Supplying cultures of microalgae and other plants with CO_2_ to increase growth is a commonly used laboratory and even industrial technique^46^. The growth promotion of *Arabidopsis thaliana* by the PGPB *Serratia odorifera* (produces quantities of volatiles) was partly attributed to enrichment with CO_2_^30^. Consequently, the significant improvement in growth from exposure of C*. sorokiniana* to volatiles of both PGPB and *E. coli* could also reasonably be attributed to the effects of CO_2_ produced by the bacteria.

The relative importance of CO_2_ in growth promotion in the experiments reported here was evaluated by reducing the quantity of CO_2_ in the headspaces of the experimental flasks using lithium hydroxide. Removal of CO_2_ in the headspace completely inhibited autotrophic growth of *C. sorokiniana* growing alone, but not when a PGPB was present in the adjacent flask. Under conditions of physical separation, growth of the microalgae was initially delayed but resumed after a few days. We suggest that this happened as the concentrations of CO_2_ produced by the bacteria increased and the filter could not absorb all the CO_2_ that was continuously produced by the PGPB. Alternatively, this was also assisted by the organic volatiles produced by the PGPB. Finally, CO_2_ can affect the pH of the microalgae substrate. CO_2_ that accumulated in the headspace of the culture can be incorporated as carbonate in the medium and consequently raise the pH to more neutral values than the initial pH, as shown in this study. Extremely low pH negatively affects growth of *Chlorella* spp.^19^. Reduction of O_2_ partial pressure in the enclosed flasks is less likely. Although theoretically this can inhibit photosynthesis and growth of microalgae, these parameters were enhanced in our system.

We have also found that the enteric bacteria *E. coli* had similar growth promotion effects on *C. sorokiniana* as both PGPB. While co-culturing *E. coli* with *C. minutissima* yielded faster growth and cell density in the microalgae^47^ and a surprising phytostimulation of maize by *E. coli* was also reported^48^, so far, no solid explanation for these effects have been provided. Consequently, we assumed that the growth effect can be partly attributed to production of large quantities of CO_2_, as well known for *E. coli*. It seems unlikely that the results of these experiments can be explained as simply due to the effects of CO_2_ enhancement, however, as *B. pumilus* had the highest CO_2_ production, but did not produce the highest growth enhancement of the microalgae during the first 72 h.

The literature on organic volatile-mediated bacterial-plant interactions (see Introduction for references) suggests that production of growth-promoting volatiles is a widespread phenomenon among rhizosphere bacteria. The presumptive effect of volatiles of *A. brasilense* was predicted^34^, but neither determined nor quantified. In contrast, the presence of *Bacillus* sp. volatiles and their effect on plant growth are well documented^28^,^29^,^49^,^50^. Both PGPB tested in our study produced a large array of volatiles, some of which are known for growth promotion, such as 2,3-butanediol and acetoin. Their role as plant growth-promoting volatile determinants has even been proven through exogenous application to plants^29^. The abundance of these two key volatiles in *A. brasilense* and *B. pumilus* volatile blends was far higher than what was previously reported for *B. subtilis* and *B. amyloliquefaciens*^28^. This comparison requires that a reservation be added. A direct comparison cannot be made because different volatile collection methods were used in these studies, namely dynamic versus static SPME headspace sampling. Additionally, using SPME fiber coatings in our study may have limited sensitivity by preferentially absorbing or excluding particular volatiles, based on polarity or size. For example, divnylbenzene/carboxen/PDMS fibers favor short-chain polar compounds such as 2,3 butanediol^51^.

In this study, as in others involving plant–volatile interactions, the biological functions for several identified volatiles were not established^26^,^52^. Some volatiles (nonanoic acid, indole, nonanal, isovaleric acid, and pentanoic acid) affect other organisms, whereas other detected mVOCs have no currently known function. In view of the chemical complexity and diversity of mVOCs, assessment of many of these compounds as relevant growth enhancers remains to be done^27^,^52^.

Both CO_2_ and organic volatiles produced by PGPB were measured in this study. CO_2_ serves as a natural substrate for growth of photosynthetic microalgae and organic volatiles have been shown to promote the growth of several plants. Our experimental design, while demonstrating that these volatiles did affect growth and metabolism of *C. sorokiniana*, could not distinguish between the relative contribution of the compounds. The extremely rapid multiplication of microalgae (seen after removal of CO_2_ by the lithium oxide filter followed by replenishment of CO_2_ by the remote presence of the PGPB) could be attributed to a starvation effect for carbon in the microalgae. Similar responses in microalgal growth rate are known after being deprived of nitrogen and phosphorus^53^.

Cells of *A. brasilense* attached to *C. vulgaris* and *C. sorokiniana* significantly promote accumulation of starch^15^,^16^, lipids^18^, fatty acids^17^, and pigments^18^ in the microalgae. Volatiles of *B. subtilis* and *E. coli* promote accumulation of starch in *Arabidopsis*^41^. As a result of the minimal mineral culture medium we used, the absolute quantity of these metabolites is lower than quantities produced when the microalgae are growing in rich medium. Still, our study clearly demonstrated that an enhancement of the production of key metabolites can be observed in the absence of any physical attachment of the PGPB to the microalgae cells. This provides insights into a novel mechanism in microalgae–bacteria interactions. This phenomenon may be complementary to the established paradigm of attachment of PGPB to plant surfaces, but can only be investigated under special culturing conditions.

The biotechnological ramifications of this study need to be explored further. Both PGPB used here are produced on a large industrial scale as agricultural inoculants^54^. *E. coli* is also used for many biotechnological applications^47^. During culturing, volatiles from these bacteria are diffused to the air and lost. The results presented here suggest a potential alternative biotechnological option. If these volatiles can be captured, they can be used to promote the performance of *Chlorella*, which is the most common economically produced genus of microalgae.

## Conclusion

This study demonstrated that diffusion of CO_2_ and organic volatile compounds produced by two PGPB promote cell growth and metabolite content in the microalgae *C. sorokiniana* without physical contact.

## Materials and Methods

### Microorganisms and initial growth conditions

The unicellular microalga *Chlorella sorokiniana* Shih. et Krauss (UTEX 2714, University of Texas, Austin, TX; formerly *C. vulgaris* UTEX 2714^55^,) and two strains of PGPB were used: *Azospirillum brasilense* Cd (DSMZ 1843; Deutsche Sammlung von Mikroorganismen und Zellkulturen, Braunschweig, Germany) and *Bacillus pumilus* ES4^56^. This microalgal strain can grow at pH 5 and higher. In experiments that measured the potential effect of CO_2_, *Escherichia coli* DH5α (Invitrogen, Carlsbad, CA) served as the negative control for microalgal growth and promoting metabolites because it does not have any plant growth-promoting effects; it also served as a positive control in the CO_2_ experiment because it produces CO_2_, as any *E. coli*. All cultures were tested under sterile conditions.

For initial culturing of the microalga, 10 mL axenic *C. sorokiniana* culture, cultivated in sterile mineral medium (C30), was added to a sterile flask containing 90 mL sterile C30 medium, composed of (in g·L^−1^): KNO_3_ (25), MgSO_4_•7H_2_O (10), KH_2_PO_4_ (4), K_2_HPO_4_ (1), FeSO_4_•7H_2_O (1), and (in μg·L^−1^): H_3_BO_3_ (2.86), MnCl_2_•4H_2_O (1.81), ZnSO_4_•7H_2_O (0.11), CuSO_4_•5H_2_O (0.09), NaMoO_4_ (0.021), pH 5.25 and incubated at 27 ± 2 °C on a rotary shaker at 120 rpm under 60 μmol photon·m^−2^·s^−1^ continuous light intensity for 6 days^57^. Initial culturing of *A. brasilense* was done in BTB medium and *B. pumilus* and *E. coli* were both cultivated in TYG medium^54^ in 125 mL flasks and incubated at 32 ± 2 °C for 16–18 h on a rotary shaker at 120 rpm. Bacteria were then harvested by centrifugation at 3720 × *g* for 7 min, rinsed twice in 0.85% saline solution, and subsequently transferred to minimal mineral Brunner’s medium (DSMZ medium 457) composed of (in g L^−1^): Na_2_HPO_4_ (2.44), KH_2_PO_4_ (1.52), (NH_4_)_2_SO_4_ (0.50), MgSO_4_•7H_2_O (0.20), CaCl_2_•2H_2_O (0.05), EDTA (0.50), FeSO_4_•7H_2_O (0.20), and (in μg·L^−1^): ZnSO_4_•7H_2_O (0.10), MnCl_2_•4H_2_O (0.03), H_3_BO_3_ (0.30), CoCl_2_•6H_2_O (0.20), CuCl_2_•2H_2_O (0.01), NiCl_2_•6H_2_O (0.02), Na_2_MoO_4_•2H_2_O (0.03). The mineral medium was supplemented with 5 g·L^−1^ glucose (for *B. pumilus* and *E. coli*) or 5 g·L^−1^ gluconate (for *A. brasilense*). Cells were grown for 48 h at 32 ± 2 °C and 120 rpm. After incubation, the bacterial cultures were rinsed, as described above, diluted in saline solution, and subsequently served as the inoculum for 250 mL flask, each containing 2 × 10^9^ CFU·mL^−1^ inoculum concentration. *C. sorokiniana* was inoculated into the same flasks using 10 mL of a suspension containing 8 × 10^6^ cells·mL^−1^.

### Experimental design and culturing conditions

**Figure 3:**
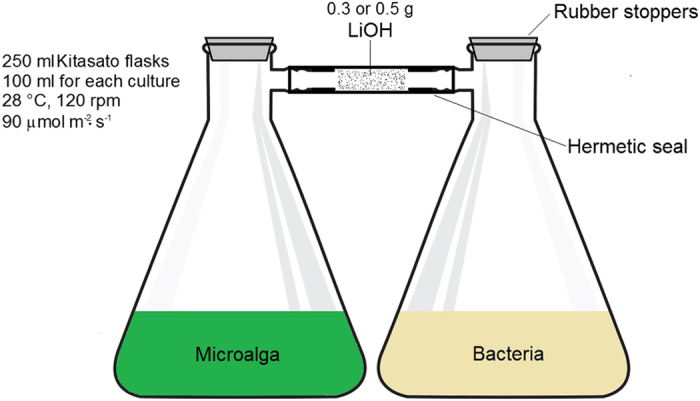
Schematic representation of the assembly of microalgae plant growth-promoting bacteria used to measure remote effect of PGPB emissions on microalgae. Lithium hydroxide filter was used only in experiments designed to remove CO_2_. In all other experiments, the filter was not used.

All experiments were run in batch cultures in pairs of 250 mL sterile Kitasato (Büchner) flasks (Corning, Corning, NY) containing 100 mL medium in each flask, with each pair of flasks serving as a single experimental unit (Fig. 3). Contents of the flasks were inoculated with the respective microorganism, in the above mentioned media, hermetically sealed with a new rubber stopper, and incubated at 28 ± 1 °C for 96 h under illumination of 90 μmol photon·m^−2^·s^−1^ on a rotary shaker at 120 rpm. Some experiments were incubated for up to 216 h. A lithium hydroxide filter (described below) was used in experiments designed to remove CO_2_. In all other experiments, the filter was not used.

Each experiment was performed with five replicates per treatment, where a pair of Kitasato flasks served as a single replicate. Each experiment contained the following treatments: *A. brasilense* and *C. sorokiniana; B. pumilus* and *C. sorokiniana, C. sorokiniana* and distilled water (as a control) and, in experiments involving potential production of CO_2_, *C. sorokiniana* and *E. coli* (as a positive control).

### Counting microorganisms

In each experiment, five samples from each flask and from each treatment were counted at each sampling period. *C. sorokiniana* cells were counted under a light microscope, using a Neubauer hemocytometer (bright line counting chamber, Hausser Scientific, Horsham, PA) connected to an image analyzer (Image ProPlus 6.3, Media Cybernetics, Silver Spring, MD). *A. brasilense* Cd, *B. pumillus* ES4, and *E. coli* DH5α were counted after serial dilution by the plate count method on nutrient agar medium (M7519, Sigma-Aldrich, St. Louis, MO). Cell volume of *C. sorokiniana* was measured by the same image analyzer. Five samples per treatment per each sampling time were analyzed and each was analyzed by five microscopic fields (n = 50 individual analyses). The volume of spherical cells was calculated.

### Analytical methods

#### Determination of total carbohydrate content

Microalgal cells extracted by centrifugation were hydrolyzed with acid for 60 min at 100 °C^16^ to release carbohydrates. Quantification of carbohydrates was by the phenol-sulphuric acid method^58^, adapted to a microplate using glucose as standard.

#### Determination of total lipid content

Extraction of lipids followed the standard method^59^ with small modifications to adapt it to microalgae, which involved sonication to disrupt cell walls^17^. Lipids were quantified in the range of 70 μg to 1.33 mg permitted by this analytical method^60^.

#### Determination of chlorophyll *a* content

Extraction of chlorophyll *a* from cells used the method described by Youngman^61^, with minor modifications, where 5 mL freshly harvested culture were centrifuged for 10 min at 6000 × *g*. The supernatant was discarded and 5 mL 90% methanol was added to the pellet and heated in a water bath for 10 min at 60 °C. After cooling, the samples were incubated in the dark for 24 h at 4 °C. Then, the samples were centrifuged for 10 min at 4 °C at 6000 × *g*. Absorbance in the supernatant was recorded at 655 and 750 nm. To quantify chlorophyll *a* content, we used the equation^61^: Chlorophyll *a* (mg·L^−1^) = [13.9 (OD_655_ – OD_750_) × U] / V, where, U is the final volume of methanol and V is the volume of the sample.

#### Determination of production of bacterial CO_2_ and reduction of CO_2_ in the headspace of the flasks

Bacterial species were cultivated on modified Brunner’s medium, as described earlier, for 48 h at 30 °C in sealed serum bottles. Concentrations of headspace CO_2_ were quantified by gas chromatography (model 8610 C, SRI Instruments, Torrance, CA) equipped with methanizer. Briefly, the CO_2_ in 100 μL injections of samples was converted to methane via the methanizer (a device for the high temperature reduction of CO_2_ to methane in the presence of a catalyst) held at 380 °C, with the methane subsequently passed via a 1 m silica gel column held at 80 °C and detected with a flame ionization detector. The gas chromatograph was calibrated using CO_2_ standards (Matheson Tri-Gas, Basking Ridge, NJ). Reduction of quantities of CO_2_ in the headspace of the flasks was accomplished by incorporating a UV-sterilized (lithium hydroxide plus water) filter, which strongly adsorbs CO_2_ (12) at quantities of 0.3 or 0.5 g per filter into culture flasks.

### Analysis of microbial volatile organic compounds (mVOCs)

Bacteria cultures were inoculated by pipetting 10 μL glycerol stock prepared in tryptic soy agar (TSA) containing 20% glycerol in 50 mL MS liquid medium^62^ containing 1.5% (w/v) sucrose, 0.4% (w/v) TSA and kept for 24 h at 37 °C. For volatile capture, 5 mL aliquots of the broth were placed in a 20 mL solid-phase microextraction (SPME) vials in a laminar flow hood. Then, 10 μL of ultra-pure (Resistivity: 18.2 MΩ·cm at 25 °C) water containing 1 μg cis-3-hexeneyl acetate (Sigma-Aldrich, St. Louis, MO) as the internal standard was added and the vials sealed withTeflon^®^-lined magnetic caps using a hand crimper to prevent the escape of volatiles. SPME and gas chromatography–mass spectrometry analysis of the volatiles were performed as detailed below^49^. Earlier studies, in which the volatile composition of media alone was determined using the same analytical approach, showed a very negligible background (i.e., Fig. 2 in Ryu *et al*.^29^).

To capture the collected volatiles, a 50/30 μm DVB-CAR-PDMS SPME fiber (57328-U, Supelco, Bellefonte, PA) was inserted into the headspace above the bacterial culture and the vials were placed in a temperature-controlled oven at 50 °C. Heating is essential and used in all SPME methods to ensure equilibration and saturation of volatiles in the headspace. Adsorption of volatiles was performed for 30 min, and fibers were desorbed at 210 °C for 1 min in the injection port of a gas chromatograph interfaced with a mass spectrometer (GC-17A GC and QP-5000 MS, Shimadzu, Kyoto, Japan).

Volatiles were separated on a DB5-MS capillary column (30 m length, 0.25 mm inner diameter, (J&W Scientific/Agilent Technologies, Santa Clara, CA). Injections were made in the split-less mode for 30 s. The gas chromatograph was operated under the following conditions: injector 220 °C, column oven 36 °C for 3 min, then programmed at a rate of 12 °C min^−1^ to 180 °C, kept at 180 °C for 5 min, and then increased by 40 °C·min^−1^ to 220 °C and held for 2 min. Helium carrier gas was injected at 1 mL·min^−1^. The transfer line and ion-source temperatures were adjusted at 230 °C and 180 °C, respectively. The quadrupole mass spectrometer (QP-5000 MS, Agilent Technologies) was operated in the electron ionization mode at 70 eV. The scan range was set at 40–500 m/z (mass-to-charge ratio). Volatile components were identified using the procedure described in Farag and Wessjohann^63^ and peaks were first deconvoluted using AMDIS software (www.amdis.net) and identified by its Kovat retention indices (RI) relative to n-alkanes (C_6_–C_20_) in the NIST/EPA/NIH mass spectral library database and with volatile standards, when available. The Kovat index refers to relative retention time measurements comparing hydrocarbon stand mixture C_8_–C_20_ to allow comparisons among databases.

### Statistical analysis

Each experiment was repeated at least twice. Results presented are the average of two or three experiments, in each case. All data was analyzed by ANOVA, employing Fisher’s post-hoc analysis at *p* < 0.05 in Statistica 8.0 software (StatSoft, Tulsa, OK).

Considering the complexity of GC-MS data, multivariate data analyses are commonly used to detect compositional differences between species and help identify potential chemical markers for discrimination in an untargeted manner. Principal component analysis (PCA) is the most commonly used unsupervised multivariate data analysis method. Models allow clustering of samples according to intrinsic variance between them and without being biased by desired outcomes^64^. Multivariate data analysis of mVOCs was done by PCA and orthogonal partial least squares-discriminant analysis (OPLS-DA), performed using the program SIMCA-P 13.0 (MKS Umetrics, Malmö, Sweden). The PCA was run to obtain a general overview of the variance of volatile metabolites, and OPLS-DA was performed to obtain information on differences in the composition of volatiles between A*. brasilense* and *B. pumilus* strains. The Distance to the Model (DModX) test was used to verify the presence of outliers and evaluate whether a sample fell within the model applicability domain.

## Additional Information

**How to cite this article**: Amavizca, E. *et al*. Enhanced performance of the microalga *Chlorella sorokiniana* remotely induced by the plant growth-promoting bacteria *Azospirillum brasilense* and *Bacillus pumilus. Sci. Rep.* **7**, 41310; doi: 10.1038/srep41310 (2017).

**Publisher's note:** Springer Nature remains neutral with regard to jurisdictional claims in published maps and institutional affiliations.

## Supplementary Material

Supplementary Information

## Figures and Tables

**Table 1:** CO_2_ production by the PGPB *Azospirillum brasilense* Cd, *Bacillus pumilus* ES4 and *Escherichia coli* and their relative growth.

	CO_2_ (mM/L)	Growth (CFUmL^−1^ × 10^8^)
*Bacillus pumilus*	133.85 ± 29.5a	5.5 ± 0.44ª
*Azospirillum brasilense*	83.56 ± 7.4a	8.02 ± 0.03b
Escherichia coli*	84	ND
Room air	12.65 ± 1.08b	ND

Different letters significantly differ at *P* < 0.05 (ANOVA and LSD post-hoc analysis for CO_2_ data and Student’s *t*-test for growth data.

^*^Kleman and Strohl^44^.

**Table 2:** Relative quantification of volatiles expressed as relative percentile and biological function of the volatile compounds produced by the three bacterial species along with their reported biological functions.

Name	Kovat index	Azospirillum brasilense		Bacillus pumilus		Escherichia coli		Biological function	Reference
		Average	SD	Average	SD	Average	SD		
Acetoin*	669	67.8	5.9	56.7	7.3	3.8	0.3	Induce growth promotion (leaf surface area), systemic resistance (ISR) and regulate auxin homeostasis in *Arabidopsis thaliana* It is an attractant to *Anastrepha ludens* (Diptera) Acetoin as a pheromone synergist for R. *palmarum*	29,65,66
2,3-Butanediol*	682	7.7	0.7	4.6	0.3	0.6	0.1	Induce plant growth promotion (leaf surface area), systemic resistance (ISR) and regulate auxin homeostasis in *Arabidopsis thaliana*.	28,29,31
α-Terpineol	1190	0.0	0.0	0.2	0.1	1.0	0.0	Antibacterial, antifungal activities, anticancer	67
(Z)- 3-Hexen-1ol, acetate (IS)	979	9.1	2.5	11.8	1.5	24.2	2.0	n/a	
***p***-methyl acetophenone	1032	0.1	0.0	0.4	0.3	0.2	0.0	n/a	
Indole*	1272	2.1	1.1	1.6	2.4	0.5	0.5	Able to regulate biofilm formation. It also induces the formation of myxospores in *Stigmatella aurantiaca*. Regulation of expression of multi-drug exporter genes and inhibition of biofilm formation of *Escherichia coli, Pseudomonas fluorescens* and *Pseudomonas aeruginosa*. Intercellular signal in microbial communities	65,68,69
***n***-Nonanoic acid	1245	0.1	0.0	0.1	0.0	0.7	0.3	Stimulation of oviposition, directing egg laying to favorable habitat of *Aedes aegypti*.	70
***p***-Dimethylbenzene	866	0.1	0.0	1.0	0.8	0.2	0.0	n/a	
Trimethyl benzene	938	0.4	0.1	2.3	1.7	0.7	0.3	n/a	
Unknown	970	0.3	0.1	2.4	1.9	0.7	0.9	n/a	
Nonanal*	1077	0.0	0.0	0.1	0.1	0.0	0.0	Active against the phytopathogenic fungus *Sclerotinia sclerotiorum*.	65
Unknown	907	0.0	0.0	0.3	0.3	0.0	0.0	n/a	
3-Carene	987	0.0	0.0	0.2	0.1	0.0	0.0	Alarm pheromone in some termite species	71
Phenylethyl alcohol	1092	1.0	0.9	0.8	0.2	0.3	0.1	Autoantibiotics produced by the fungus Candida albicans	72
Ethyl decanoate	1359	0.5	0.5	0.0	0.0	0.0	0.6	n/a	
Ethyl dodecanoate	1563	0.2	0.2	0.0	0.0	0.0	0.0	n/a	
Ethyl octanoate	1167	0.2	0.2	0.0	0.0	0.0	0.0	n/a	
***n***-Heptanoic acid	1049	0.0	0.0	0.1	0.0	0.0	0.0	n/a	
Undecane*	1071	0.0	0.0	0.2	0.1	0.0	0.0	n/a	
Dodecane*	1193	0.1	0.0	0.3	0.2	0.1	0.0	n/a	
Tridecane*	1289	0.1	0.0	0.3	0.1	61.5	12.4	Defensive against predators by the stink bug *Cosmopepla bimaculata*	73
Pentadecane*	1479	0.1	0.0	1.3	1.1	0.3	0.0	n/a	
Octadecane*	1787	0.4	0.3	0.7	0.6	0.2	0.0	n/a	
*n*-Dodecanal	1377	0.1	0.0	0.2	0.1	0.3	0.4	n/a	
2-Methylbutyric acid	840	0.6	0.2	0.8	0.3	0.0	0.0	n/a	
ButanoI 3methyl-acetate	942	0.1	0.0	0.1	0.0	0.1	0.0	n/a	
2-Decanol	1145	0.2	0.0	0.3	0.1	0.3	0.2	n/a	
Isovaleric acid	833	4.3	1.1	2.5	1.5	0.0	0.0	Stimulation of spore germination of *Agaricus bisporus* Inhibition of proliferation and cytokine production in lymphocyte cells Reduction of heat resistant spores, prevention of spore formation	74
Acetic acid*	610	0.4	0.4	3.0	1.0	0.0	0.0	Highly attractive to Mexican fruit flies Reduction of heat resistant spores, prevention of spore formation	65
***n***-Caprylic acid	1140	0.8	0.1	0.6	0.2	0.5	0.4	Decrease yeast viability	75
Acetone*	775	0.1	0.0	0.0	0.0	0.0	0.0	Inhibited growth of fungi. Has no effect on bacteria	74
2-Methylpentanal	1128	0.1	0.0	0.1	0.0	0.1	0.0	n/a	
2-Ethylhexanol	859	0.1	0.0	0.1	0.1	0.1	0.3	n/a	
Unknown alcohol	1003	0.3	0.0	1.3	0.9	0.8	0.4	n/a	
1-Hexene, 4methyl-	999	0.1	0.0	0.4	0.3	0.3	0.1	n/a	
Caproic acid	961	0.5	0.2	1.4	0.8	0.4	0.2	n/a	
Ethyl caprylate	1167	*						n/a	
Unknown furfural	997	0.8	0.2	0.4	0.1	0.0	0.0	n/a	
Unknown terpene	1224	0.2	0.2	0.8	0.3	0.1	0.0	n/a	
Unknown hydrocarbon	1278	0.3	0.3	0.2	0.1	0.3	0.2	n/a	
Unknown hydrocarbon	1360	0.2	0.1	0.5	0.2	0.4	0.3	n/a	
Unknown	1020	0.2	0.1	0.2	0.1	0.0	0.0	n/a	
Unknown	1337	0.2	0.1	1.8	1.5	0.0	0.0	n/a	

Compounds denoted by an asterisk were confirmed with authentic standard.
