# Supplementary material for: Enhanced performance of the microalga Chlorella sorokiniana remotely induced by the plant growth-promoting bacteria Azospirillum brasilense and Bacillus pumilus
Source: Sci Rep. 2017 Feb 1;7:41310. doi: 10.1038/srep41310 (PMC5286510; doi:10.1038/srep41310)
Supplement: Supplementary Information [file srep41310-s1.pdf]

Revised: Scientific Reports-Nature

Enhanced performance of the microalga *Chlorella sorokiniana* remotely induced by the plant growth-promoting bacteria *Azospirillum brasilense* and *Bacillus pumilus*

Edgar Amavizca, Yoav Bashan, Choong-Min Ryu, Mohamed A. Farag, Brad M. Bebout, and Luz E. de-Bashan

Supplementary Figures

Suppl. Fig. S1

SPME gas chromatograms of volatiles from bacterial strain: *Azospirillum brasilense* (AB), *Bacillus pumilus* (BP) and *E. coli* (DH5α).

Peaks: 1 = acetoin; 2 = 2,3-butanediol; 3= indole. IS=internal standard.

Suppl. Fig. S2

PCA analysis of volatiles of bacterial species using SPME. (**A**) PCA plot of PC1 and PC2 scores (n = 3). (**B**) PCA loading plot for contributing peaks of PC1/PC2 and their assignments, with each volatile denoted by its mass/retention time pair value.

(●), DH5α, (■, BP; (▲AB

Suppl. Fig. S3

(**A**) OPLS-DA score plot and (**B**) loading S-plots derived from *A. brasilense* strain modelled against *Bacillus pumilus* sample. The S-plot shows the covariance p [1] against the correlation p(cor) [1] of the variables of the discriminating component of the OPLS-DA model. Cut-off values of $P < 0.05$ were used; selected variables are highlighted in the S-plot with m/z retention time in seconds. Identifications are discussed in the text.

Suppl. Fig. S4

MS spectra of the major volatile peaks: 1, acetoin (A); 2, 2,3-butanediol, (B); and 3, indole (C) showing for each peak its MS spectrum plotted against authentic spectrum in NIST database and with a match score quality above 900 as highlighted in red bar. Volatile structure is depicted inside MS spectra window. In all cases, reported values represent the percentage of total volatiles detected normalized to the

amount of the internal standard hexneyl acetate. No absolute measurements of individual volatiles were made. This followed a similar approach done with plants [45].

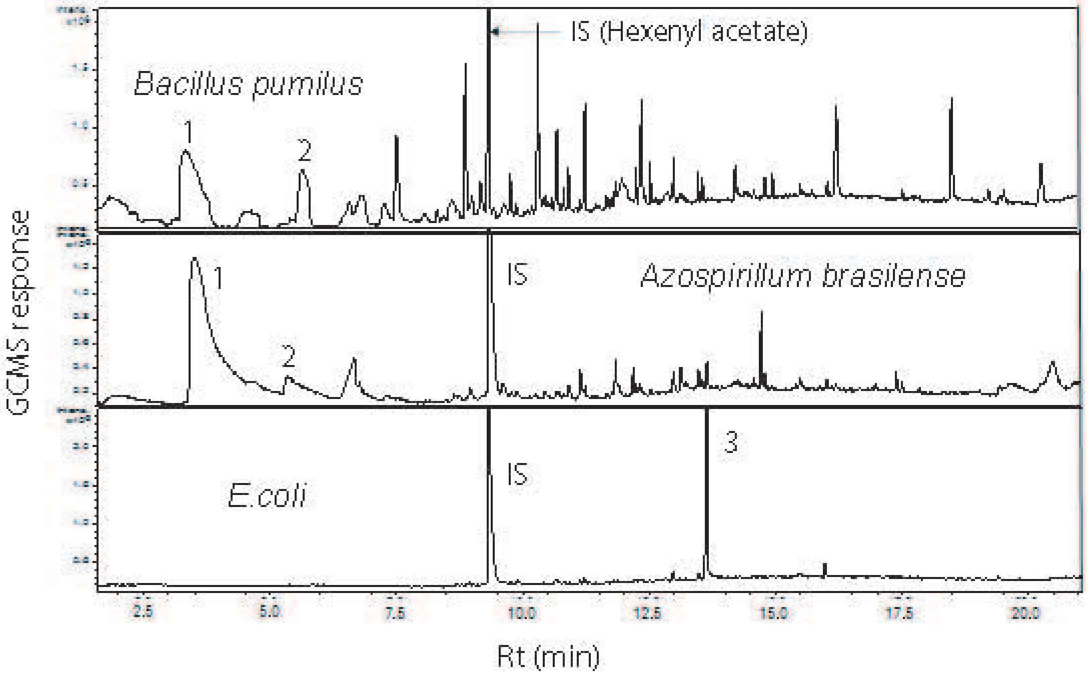

IS (Hexenyl acetate)
Bacillus pumilus
1
2
IS
Azospirillum brasilense
1
2
IS
3
E.coli
GCMS response
2.5
5.0
7.5
10.0
12.5
15.0
17.5
20.0
Rt (min)

Multivariate data analysis of mVOCs dataset

Although differences were apparent by visual examination of the GC-MS chromatograms of the different species (Suppl. Fig. S1), multivariate unsupervised data and supervised analysis, including PCA and OPLS, were adopted to assess heterogeneity between different bacterial strains in an untargeted approach. We used PCA to summarize the multivariate variation for the data matrices in GC-MS fingerprinting analyses. For the volatile profiles of all the samples, the first two principal components (67%, 18%) were significant and accounted for more than 90% of the total variance. The scores and loading plots of the first two principal components (Suppl. Fig. S2A) showed differentiation of strains into two groups. On the left side of the plot, samples of *A. brasilense* and *B. pumilus* (negative PC1) clustered close together; *E. coli* was located on the left side (positive PC1). The metabolite loading plot for PC1 (Suppl. Fig. S2B), which presents the most important components with respect to scattering behavior, showed that acetoin and 2,3-butanediol, CO_2, and indole have more influence on discrimination of the samples, with *A. brasilense* and *B. pumilus* releasing higher levels of acetoin and 2,3,butanediol vs. CO_2, and indole enrichment in *E. coli* volatile blend.(Table 1).

In spite of clear separation by PCA for bacterial strains, based on its species type, *B. pumilus* could not be distinguished from *A. brasilense* (Suppl. Fig. S2A). PCA was efficient for distinguishing PGPR strains with different volatiles profiles (Farag et al 2006). Consequently, supervised OPLS-DA was used to build a classification model that would distinguish between the bacteria. OPLS-DA has greater potential in identifying markers by providing the most relevant variables for differentiation between two sample groups. *A. brasilense* was modelled against *B. pumilus* using OPLS-DA, with the derived score plot showing a clear separation between both samples (Suppl. Fig. S3A). The OPLS score plot explained 96% of total variance

(R^2 = 0.96), with the prediction goodness parameter Q^2 = 0.99. A particularly useful tool that compares the variable magnitude against its reliability is the S-plot obtained by the OPLS-DA model and represented in Suppl. Fig. S3B, where axes plotted from the predictive component are the covariance p[1] against the correlation p(cor)[1]. For plots with retention time *m/z* values, a cut-off value of $P < 0.05$ was used. Compared with *B. pumilus, A. brasilense* is enriched in acetoin and 2,3-butanediol. Most interestingly, these mVOCs results parallel growth promotion effect observed in Figs. 2 A, D, with *A. brasilense* showing slightly stronger effect than *B. pumilus*.

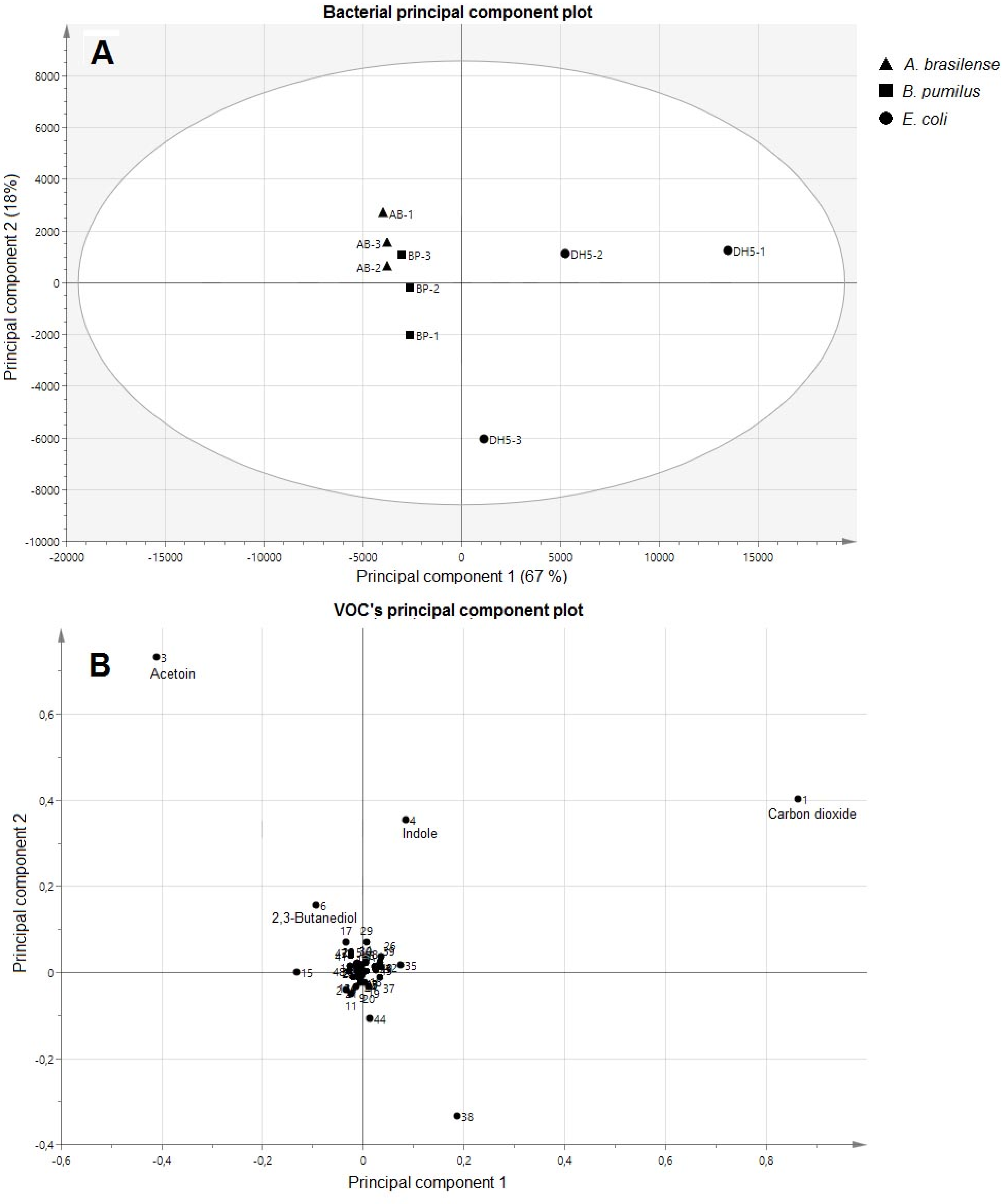

Bacterial principal component plot
A
A. brasilense
B. pumilus
E. coli
AB-1
AB-3
AB-2
BP-3
BP-2
BP-1
DH5-2
DH5-1
DH5-3
Principal component 2 (18%)
Principal component 1 (67 %)
8000
6000
4000
2000
0
-2000
-4000
-6000
-8000
-10000
-20000
-15000
-10000
-5000
0
5000
10000
15000
VOC's principal component plot
B
3
Acetoin
1
Carbon dioxide
4
Indole
6
2,3-Butanediol
17
29
26
39
35
15
48
37
11
20
44
38
Principal component 2
Principal component 1
0,6
0,4
0,2
0
-0,2
-0,4
-0,6
-0,4
-0,2
0
0,2
0,4
0,6
0,8

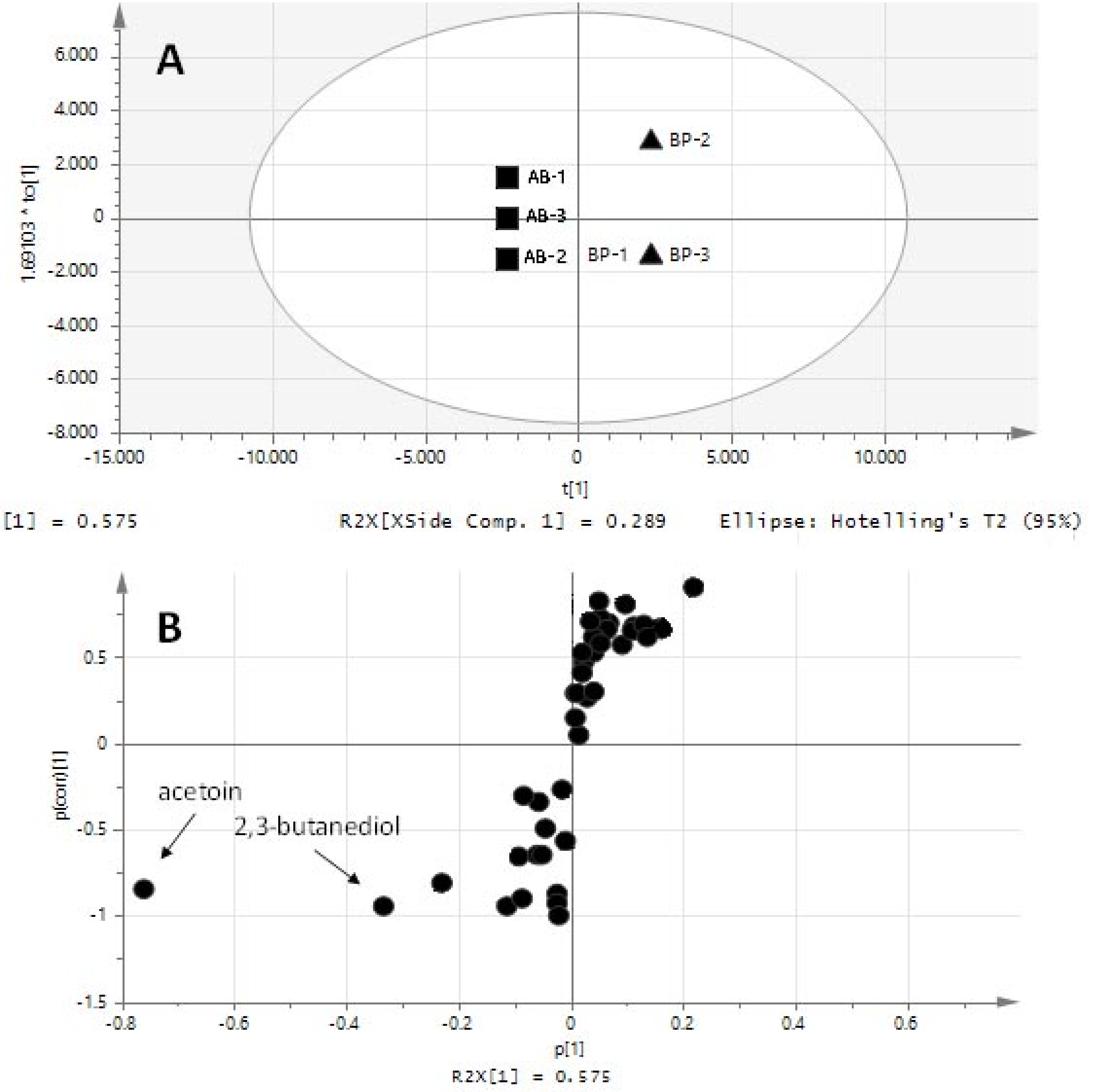

A
BP-2
AB-1
AB-3
AB-2
BP-1
BP-3
t[1]
[1] = 0.575
R2X[XSide Comp. 1] = 0.289
Ellipse: Hotelling's T2 (95%)
B
acetoin
2,3-butanediol
p[1]
p(corr)[1]
R2X[1] = 0.575

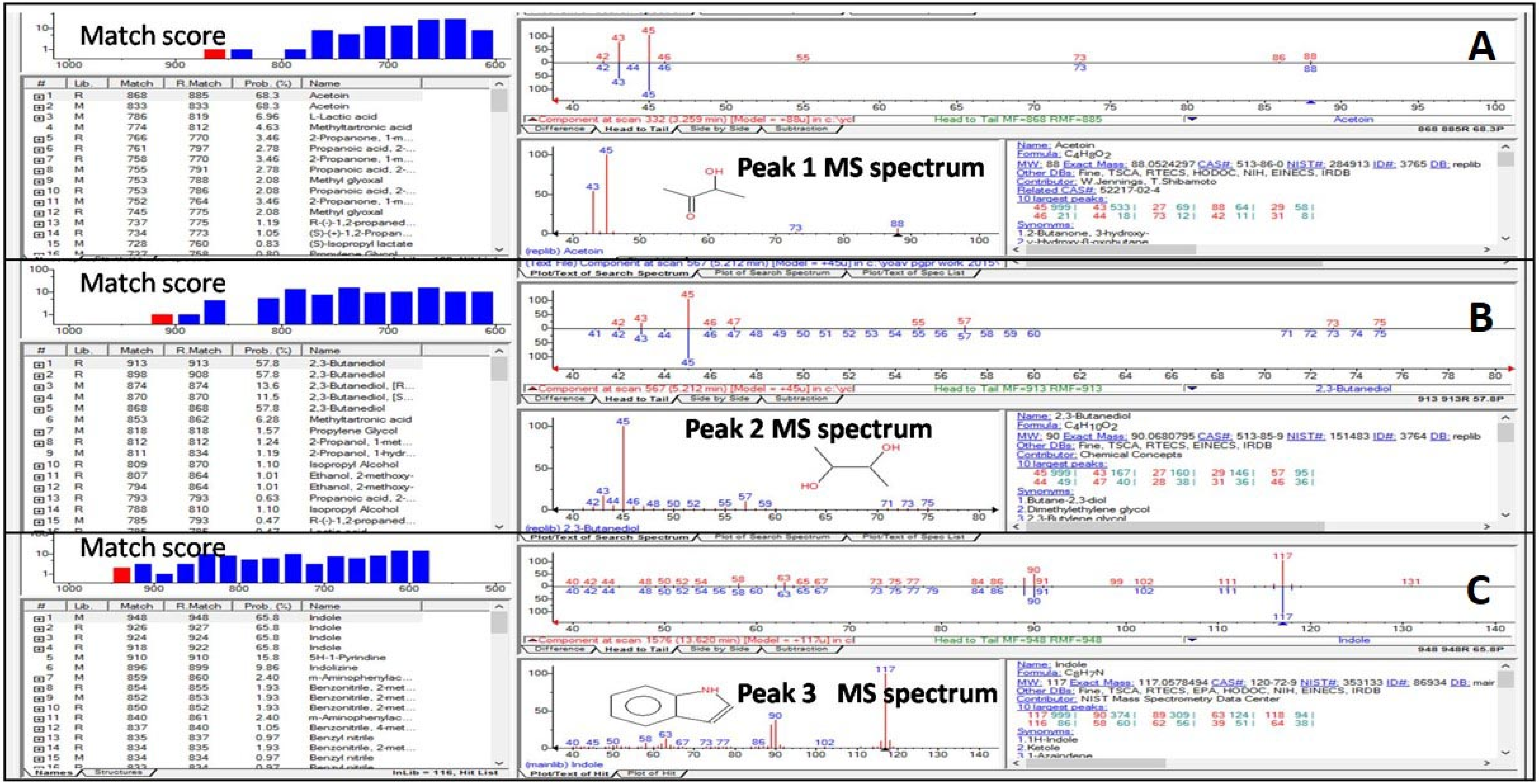

Match score
A
Peak 1 MS spectrum
Match score
B
Peak 2 MS spectrum
Match score
C
Peak 3 MS spectrum
